# Effects of Cross-Linking on Physicochemical and Film Properties of Lotus (*Nelumbo nucifera* G.) Seed Starch

**DOI:** 10.3390/foods11193069

**Published:** 2022-10-03

**Authors:** Ankita Chandak, Sanju Bala Dhull, Sneh Punia Bangar, Alexandru Vasile Rusu

**Affiliations:** 1 Department of Food Science and Technology, Chaudhary Devi Lal University, Sirsa 125055, India; 2 Department of Food, Nutrition and Packaging Sciences, Clemson University, Clemson, SC 29631, USA; 3 Life Science Institute, University of Agricultural Sciences and Veterinary Medicine Cluj-Napoca, 400372 Cluj-Napoca, Romania; 4 Animal Science and Biotechnology Faculty, University of Agricultural Sciences and Veterinary Medicine Cluj-Napoca, 400372 Cluj-Napoca, Romania

**Keywords:** lotus seed starch, cross-linking, edible film, properties

## Abstract

Lotus seed starch was cross-linked using sodium trimetaphosphate (STMP) in varying amounts (1, 3, and 5%), and its rheological, pasting, thermal, and physicochemical properties were investigated. These cross-linked lotus seed starches (CL-LS-1, CL-LS-3, CL-LS-5) were also used to produce films (CL-LSFs), which were then examined for their mechanical characteristics, water vapor permeability, moisture content, opacity, thickness, and water solubility. After cross-linking, the solubility, amylose content, and swelling power of all the starch samples decreased. Cross-linking resulted in an increased pasting temperature, while peak viscosity (PV) decreased, with CL-LS-5 exhibiting the lowest peak viscosity (1640.22 MPa·s). In comparison to native starch, the thermal characteristics of CL-LS demonstrated greater gelatinization temperatures (T_o_, T_p_, T_c_) and gelatinization enthalpy (ΔH_gel_). The gelatinization enthalpy of CL-LS varied between 152.70 and 214.16 J/g, while for native LS the value was 177.91 J/g. Lower moisture content, water solubility, and water vapor permeability were observed in the CL-LSFs. However, the cross-linking modification did not produce much effect on the film thickness. The highest tensile strength (12.52 MPa) and lowest elongation at break (26.11%) were found in CL-LSF-5. Thus, the starch films’ barrier and mechanical qualities were enhanced by cross-linking.

## 1. Introduction

In the last century, plastic-based packaging material has found great application in the food and non-food sector owing to its excellent properties. However, the worldwide production of thousands of tonnes of plastic waste every year is of serious concern [1]. Globally, at the present rate of plastic use and disposal, nearly 12,000 Mt of waste is estimated to be deposited in the environment by 2050 [2,3]. Several adverse environmental concerns, such as limited biodegradability leading to long-term accumulation and carbon dioxide (CO_2_) emission and hazardous ash/slag generation due to the incineration of plastic waste, have increased the interest in developing alternative biodegradable packaging materials [4]. Recently, many polymers of biological origin have been suggested and investigated to produce biodegradable films. Starch is the most explored polymer due to its abundance, biodegradability, low cost, renewability, well-explored history of properties, and modification strategies [5]. It has a wide range of industrial uses, including food, pharmaceuticals, paper, film formation, packaging, and textiles. It is used as the main constituent in many starch-rich foods such as pasta, noodles, and bread [6]. Moreover, edible films made from starches are colorless, non-toxic, odorless, tasteless, and semi-permeable to O_2_, moisture content, lipid, and CO_2_, along with flavor components [7]. However, conventional starch sources such as corn, rice, potato, etc., are becoming less available due to catering to the food requirements of the increasing population [5]. Therefore, the need for novel starch sources with better functional properties has risen today. In the present study, lotus seed starch (LS), a non-conventional starch with high amylose content (30–50%) suitable for film forming but which has not been utilized to date for the preparation of biodegradable films [8], was selected to explore its potential.

Lotus (*Nelumbo nucifera* G.), also known as Chinese water lily, Indian lotus, and sacred lotus, belongs to the family *Nelumbonaceae* and is mostly grown in Australia, Japan India, Thailand, China, and South Korea [9,10]. Every part of the plant, including rhizomes, pods, seeds, and leaves, are utilized for food and pharmaceutical purposes in China and India. The starch content of lotus seeds is nearly 50%, with a high amylose content (40%) and granules of varying shape and size [11]. LS, like other starches, is used in different food and non-food applications as a thickener, filler, gelling agent, stabilizer, and water-binding agent [12]. However, native LS has limited applications due to its lower solubility, transparency, faster retrogradation, and poor refrigerated storage stability [13,14].

The starch properties can be tailored in a desired manner using different modification techniques. Of these techniques, chemical modification, such as cross-linking using chemical agents (e.g., epichlorohydrin, sodium trimetaphosphate (STMP), and sodium tripolyphosphate (STPP)), can be a promising method that modifies starch properties by introducing new bonds [15]. Through bridging, these chemical bonds strengthen the intermolecular H-bonding for connecting polymer chains to form a three-dimensional network [16]. In the present study, STMP was used as a cross-linker for LS due to its non-toxic nature, lower penetration rate, and promising cross-linking ability [17]. The cross-linking of starches decreases the amylose content, while viscosity, swelling capacity, shear resistance, and freeze–thaw stability is improved due to their stability at higher temperatures and their low retrogradation tendency [18].

In the packaging sector, the replacement possibility of synthetic/plastic material from biopolymers such as starch, cellulose, etc., in film preparation has created many opportunities today [19,20]. However, a serious challenge to the commercialization of native starch film is its poor mechanical strength and high solubility. However, due to the incorporation of new covalent bonds with pre-existing H-bonds, cross-linking results in the improved mechanical, thermal, and acid tolerance of modified starches. As a result, the tensile strength, solubility, and opacity of biodegradable starch films increases while water vapor permeability decreases after STMP cross-linking [21]. Our group recently reviewed the different properties and modifications of LS and its application history [8], finding that no information is available on STMP cross-linked LS and its use in the development of films. Thus, the present study was planned to study the effect of different levels of STMP cross-linking (1, 3, 5%) on the physicochemical and film-forming properties of LS. Moreover, the mechanical and selected barrier properties of native and modified LS films were explored for their possible food packaging applications.

## 2. Materials and Methods

### 2.1. Materials

Lotus seeds were procured from the local market in Sirsa, Haryana, India. All the chemical reagents used were of analytical grade.

### 2.2. Starch Isolation

The starch was isolated from lotus seeds using the method described by Punia et al. [11], with some modification. The kernels of lotus seeds were removed from the seed coats after breaking them open. The kernels were cut and pulverized with ice-cold water, and the resulting slurry was screened through cheesecloth. The fibrous residue was further homogenized and squeezed to release the starch trapped inside. The slurry was filtered through sieves (0.250, 0.150, 0.100, 0.075, 0.045 mm) and left to settle for 4–5 h. The settled starch layer was resuspended in distilled water and centrifuged at 3200× *g* for 10 min, and the supernatant was withdrawn. To purify starch, the sediment was washed five times and the final sediment was hot-air-dried (45 °C, 24 h). After drying, LS was pulverized and sieved through a 100-mesh sieve, and the fine powder was stored for further analysis.

### 2.3. Cross-Linking of Lotus Seed Starch

The cross-linked lotus seed starch (CL-LS) was prepared using the method described by Woo and Seib [22]. We mixed 100 g of LS with 250 mL distilled water, to which STMP (1.0%, 3.0%, and 5.0%) was added, and the pH was adjusted to 10.5 by using a 5% NaOH solution. After continuous stirring (at 45 °C/1 h), the reaction was stopped by adjusting pH to 5.5 using 2% HCl. Then, we washed to neutralize, centrifuged (3000× *g*, 10 min), and dried (45 °C/24 h) the CL-LS samples. The samples were marked as native LS, CL-LS-1, CL-LS-3, and CL-LS-5 for 0, 1, 3, and 5% STMP levels, respectively.

### 2.4. Properties of Native and Cross-Linked Lotus Seed Starch

#### 2.4.1. Degree of Cross-Linking

The degree of cross-linking of modified LS was measured using the method published by Kaur et al. [23]. A rheometer (MCR-52, Anton Paar, Graz-8054, Austria) with an in-built starch cell was used to examine the degree of cross-linking of CL-LS samples. An aqueous suspension of CL-LS (15%) was equilibrated for a period of one minute at 50 °C and then heated from 50 to 95 °C at 11 °C/min, and then held at 95 °C for 2.7 min. The suspension was then cooled to 50 °C at the same rate and held for 2 min (at 50 °C). It was measured using the following Equation (1).

(1) $$\mathrm{Degree}\;\mathrm{of}\;\mathrm{cross}-\mathrm{linking}=\frac{X-Y}{X}\;\times \;100$$

where *X*: PV of native LS, and *Y*: PV of CL-LS samples.

#### 2.4.2. Amylose Content

The amylose content of LS was measured using the method published by Williams et al. [24], with minor modifications. The absorbance of the starch solution was noted at 625 nm using a UV-VIS spectrophotometer (Thermo Scientific, Genesys 10S UV-VIS, Ahmadabad, India). A standard curve made from amylopectin and amylose blends was used to determine the amylose content in triplicate.

#### 2.4.3. Swelling Power and Solubility

The swelling power (SP) and solubility (S) of LS were measured using the method published by Leach [25], with minor modifications. Initially, 1 g of LS was mixed with 99 mL distilled water, heated (90 °C, 1 h), cooled in an ice-water bath for 1 min, equilibrated (25 °C, 5 min), and centrifuged (3000× *g*, 30 min). The supernatant was then poured into a preweighed Petri plate, dried, cooled, and weighed again. The weight of swollen starch sediment was also noted. The swelling power (SP) and solubility (S) were determined with the following Equations (2) and (3):

SP (g/g) = Weight of sediment (g)/Weight of dry LS (g)(2)

Solubility (%) = 100 × (Wt of dried supernatant)/Wt of dry LS(3)

#### 2.4.4. Transmittance (%)

The transmittance of LS samples was determined using the method of Perera and Hoover [26]. Briefly, LS aqueous suspension (1%) was heated in a water bath at 90 °C for one hour with continuous stirring, then cooled (30 °C, 1 h) and kept refrigerated (4 °C) for five days. Every 24 h, the absorbance was determined against a water blank at 640 nm to calculate the transmittance.

#### 2.4.5. Crystalline Structure (XRD)

The X-ray diffractometer (Rigaku Miniflex, Japan) was operated at a 45-kilovolt voltage and a 40-milliampere current. The diffraction data of native LS and CL-LS were collected over an angle of diffraction (2θ), 10° to 50°. The crystallinity was calculated using Equation (4).

Crystallinity (%) = (Area of diffraction peak)/(Total area) × 100(4)

#### 2.4.6. Pasting Properties

A rheometer (MCR-52, Anton Paar, Graz-8054, Austria) with an in-built starch cell was used to examine the pasting properties of LS samples. The starch suspension (1.2 g LS + 13.8 g distilled H_2_O) was equilibrated at 50 °C for one minute and then heated from 50 to 95 °C at 6°C/minutes, and then held at 95 °C for 2.7 min. The suspension was then cooled to 50 °C at the same rate and held for 2 min (at 50 °C). The pasting viscosities (peak viscosity (PV), breakdown viscosity (BV), trough viscosity (TV), setback viscosity (SV), and final viscosity (FV)) and pasting temperature (PT) were measured from the pasting graph.

#### 2.4.7. Dynamic Rheological Behavior

The frequency sweep tests of all LS samples were performed using the method described by Kaur and Singh [27], with few modifications. The slurry of starch was made with distilled H_2_O at 15% *w*/*w* concentration, stirred manually, heated (90 °C, 30 min), and stirred again for 3 min. After cooling at room temperature and loading on the rheometer ram, the frequency sweep measurements were performed at 25 °C from 0.1 to 100 rad/second using the rheometer (MCR-52, Anton Paar, Graz-8054, Austria) equipped with a parallel-plate system (4 cm diameter). The G′ (storage modulus), G′′ (loss modulus), and tan δ (loss tangent) were recorded at an angular frequency of 6.28 rad/s.

### 2.5. Preparation of LS Films

LS films (LSFs) were made using the procedure reported by Dhull et al. [5], with little modification. Initially, 4 g of the starch sample was dispersed in distilled water and 2 g of glycerol was added to it while stirring continuously. Then, the solution was first heated (80°C, 20 min) in a water bath (Brookfield, TC-202, Harlow, UK) to gelatinize the starch, with periodic stirring to avoid lump formation. After cooling to room temperature, the gelatinized starch solution was passed through a muslin cloth, and the resultant solution was cast on a Teflon-coated baking sheet. The casted starch solutions were dried for 24 h at 45°C in an oven (NSW-144, New Delhi, India). The dried films were peeled off and kept at 25 °C with a 50% relative humidity (RH) for subsequent analysis. The films prepared with native LS, CL-LS-1, CL-LS-3, and CL-LS-5 were marked as: native LSF, CL-LSF-1, CL-LSF-3, and CL-LSF-5, respectively.

### 2.6. Properties of LS Films

#### 2.6.1. Moisture Content and Thickness

The moisture content of LSFs was determined gravimetrically in a hot air oven (NSW-144, New Delhi, India) at 100 °C for 24 h. The results were calculated in %. The thickness of films was analyzed using a digital micrometer with an accuracy of ±001. It was measured with 10 folds at 10 random locations and calculated in mM.

#### 2.6.2. Water Vapor Permeability (WVP) of LS Film

The water vapor permeability (WVP) of LSFs was analyzed by following the E96/E96M ASTM standard method [28]. LSFs were sealed over a cup mouth containing anhydrous calcium chloride (50 g) at 25 °C. The cell was placed inside a desiccator that was maintained at 75% RH using saturated NaCl solution. The weight gain of each cup with LSFs was noted after twenty-four hours. The water vapor transmission rate (WVTR) was calculated using Formula (5) given by Sukhija et al. [29] as follows:

(5) $$\mathrm{WVTR}=\frac{G}{\mathrm{tA}}$$

where G: weight-gain, t: time in seconds (s), and A: area of cup mouth (test area, m^2^).

WVP was measured with the help of Equation (6) given below [29]:

(6) $$\mathrm{WVP}=\frac{\mathrm{WVTR}}{S\left(R_{1}-R_{2}\right)}\times d$$

where WVP: water vapor permeability (g·m/Pa·s·m^2^); d: LSF thickness in millimeters; S: saturation vapor pressure of water in pascal at the test temperature 30 °C; and R_1,_ R_2_: percentage relative humidity in the humidity chamber and cup.

#### 2.6.3. Water Solubility

The water solubility of LSFs was analyzed using a method given by Romero-Bastida et al. [30], with little modification. Dried LSF weight was directly measured, and the films were then submerged in 80 mL distilled H_2_O at 25 °C for 24 h. The separated film samples were first dried in an oven at 60 °C until they reached a consistent weight. The following Equation (7) was used to calculate water solubility [26]:

(7) $$\mathrm{Water}\;\mathrm{solubility}\;(\%)=\frac{\left(Wi-Wf\right)}{Wi}\times 100$$

where *Wi*: initial weight of LSF in g, and *Wf*: final weight of LSF in g.

#### 2.6.4. Mechanical Properties

Tensile strength (TS) and elongation at break (percent) (EB) of native LSFs and CL-LSFs were measured using a texture analyzer (TA-X2, Stable Micro Systems, Surrey, UK) with fixed grips, according to the reference method [31]. Before testing, the LSFs were split into strips (10 mm × 50 mm). Crosshead speed and grip separation were adjusted to 1.0 mm/s and 20 mm, respectively, at the start.

#### 2.6.5. Opacity of LS Films

An empty cuvette was utilized as a control, with the LSFs cut into strips and affixed to it. At 600 nanometers, the opacity of LSFs was studied, and the following equation was used to calculate the opacity described by [32]:

(8) Opacity= A600X

where “*X*”: the thickness of LS film in millimeters, and A600: absorbance of LSF taken at 600 nm.

#### 2.6.6. FTIR Spectral Analysis

LS film samples were examined using an attenuated total reflection Fourier transform infrared spectrometer (ATR-FTIR, Bruker ALPHA 200229, Bremen, Germany). The transmittance mode of LS films was recorded in the wavelength range of 450−4000 cm^−1^. This was repeated for each LSF at least three times to obtain the spectra in terms of transmission.

#### 2.6.7. Granular Morphology (SEM)

The morphology of LSFs was analyzed using a scanning electron microscope (JSM-6100, Jeol, Peabody, MA, USA) at an accelerating potential of 10 kV with 200× magnification.

#### 2.6.8. Thermal Properties (DSC)

The thermal properties of native and modified LS films were determined using a differential scanning calorimeter (DSC). Each LS film was fractured into tiny pieces and loaded into a sample pan. An empty aluminum pan was used as a reference. The sample pan was then heated from 20 to 250 °C at a rate of 10 °C/min. The gelatinization enthalpy and gelatinization temperature (∆H_gel_, onset T_o_, peak T_p_, and conclusion T_c_) were determined automatically.

### 2.7. Statistical Analysis

The data presented in the tables were analyzed in triplicate, and single-factor analysis of variance (ANOVA) was used for the experimental results. Duncan’s multiple range tests (DMRT) using SPSS 20 (IBM Analytics, IBM Corporation, Armonk, NY, USA) were used to determine the significant (*p* ≤ 0.05) differences between the data.

## 3. Results and Discussion

### 3.1. Native and Cross-Linked Lotus Seed Starch Properties

#### 3.1.1. Determination of Amylose Content (AC) and Degree of Cross-Linking (DC) of LS

The amylose content is a primary quality characteristic of starches which governs their different properties and ultimate end-use purposes. Various methods were used to determine the amylose content in LS, including iodine-binding chromatography and by using a differential scanning calorimeter, but iodine-binding spectrophotometry remains the most common. Native LS showed an AC of 27.98% (Table 1). After cross-linking, the AC values decreased significantly (*p* ≤ 0.05) with increasing STMP concentration from 1% to 5% and ranged between 13.45 and 25.16%, with the lowest (13.45%) observed for CL-LS-5. The results are in agreement with Sharma et al. [33] who observed a similar reduction in AC for STMP cross-linked faba bean starch. The reduction in values of AC of CL-LS might be due to the intramolecular and intermolecular interactions within amylose molecules or between amylose and amylopectin molecules [34]. On the other hand, the degree of cross-linking (DC) in CL-LS at different levels of STMP (1, 3, and 5%) varied from 2.58 to 45.84%, and it increased manyfold with every increase in the level of the cross-linking agent.

#### 3.1.2. Solubility (S) and Swelling Power (SP) of LS

Swelling power (SP) describes the capability of starch granules to trap and hold water within their structure and depends more so on the amylopectin content of starches than their amylose content. The leaching of amylose and amylopectin from starch granules, followed by maximum swelling, determines starch solubility [35]. The SP and solubility of starch are indicators of the degree of interaction between starch chains within the amorphous and crystalline domains [36]. With the rise in temp. from 60 to 90 °C, there was a progressive rise in solubility and swelling power for native LS as well as CL-LS (data not shown). The solubility and swelling power of the native LS and CL-LS at 90 °C are shown in Table 1. LS swelling power varied between 9.05 and 15.02 g/g; when STMP concentration increased, swelling power decreased. The decrease in SP prevented the bursting of starch granules, which can be beneficial in improving the textural quality of foods upon cooking. It is also noted that the cross-linking process enhances the intra- and intermolecular interactions between amylose and amylopectin, hence limiting the swelling of starch molecules. These results are in accordance with Sharma et al. [33] for faba bean starch.

The solubility of LS ranged between 15.13 and 16.00%, with the highest value observed for native LS. The solubility showed a slightly decreasing trend with every increase in the concentration of STMP. CL starches showed lower solubility in comparison to their native counterpart, which was attributed to the enhanced integrity and bonding of starch granules due to the introduction of phosphate groups in CL-LS that tightly held the starch molecules. It has also been established that the density of CL starches increases in the presence of a CL agent, resulting in lower solubility due to lesser breakdown of starch granules upon gelatinization [37]. The results are in agreement with Liu et al. [38] for corn starch. As a result, both the SP and solubility of CL-LS were reduced.

#### 3.1.3. Light Transmittance

**Figure 1:**
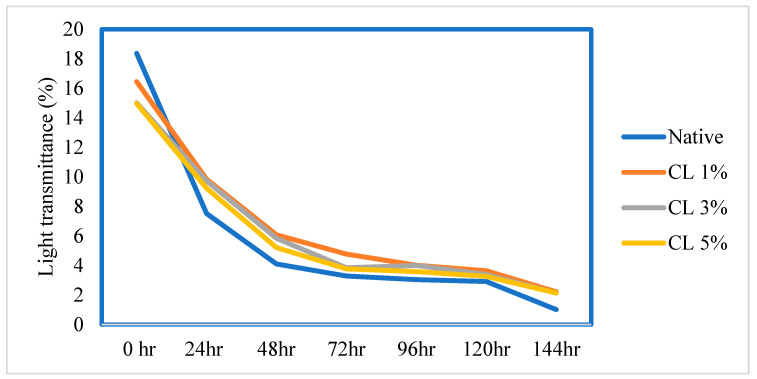
Effect of storage duration on the transmittance (%) of native and cross-linked (CL) lotus seed starch.

The change in transmittance of all LS samples is depicted in Figure 1. The part of incident light that passes through the starch at a specific wavelength is known as light transmittance. It provides information on how starch paste behaves when light travels through it. A high transmittance value shows high paste clarity, a desirable characteristic of starch. The light transmittance of LS was gradually decreased when the quantity of STMP increased, and the native starch paste was found to have the maximum value (18.57%), whereas the paste modified by CL 5% had the lowest value (14.98%). Native starch molecules disintegrate completely during gelatinization, but cross-linked starch molecules remain intact [23,39]. This resulted in a decrease in the paste clarity of the CL-LS paste.

During storage, the values for the light transmittance of the native LS and CL-LS samples (1, 3, and 5%) ranged from 1.02 to 18.57%, 2.22 to 16.45%, 2.14 to 15.02%, and 2.14 to 14.98%, respectively. It was seen that the paste clarity values for all the LS samples declined over the period of storage. This reduction might be due to starch retrogradation, which involves reassociating broken bonds in an ordered structure. Modified CL-LS had more distinct transmittance patterns than native LS paste. Light transmittance in CL-LS decreased gradually with storage; however, with native LS, a rapid drop was seen after 24 h of storage.

#### 3.1.4. Crystalline Structure

**Figure 2:**
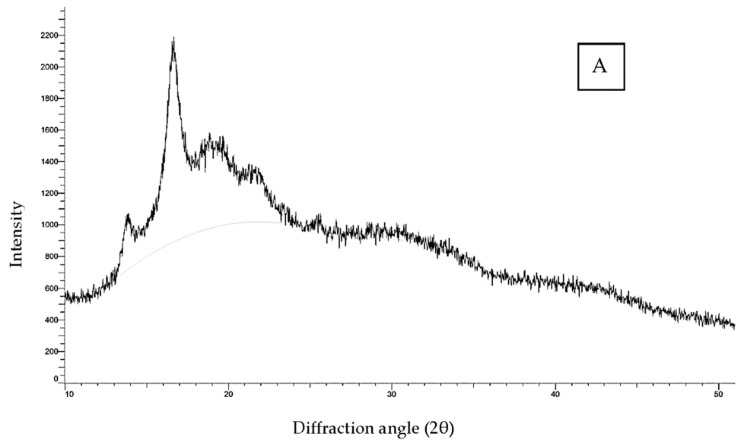

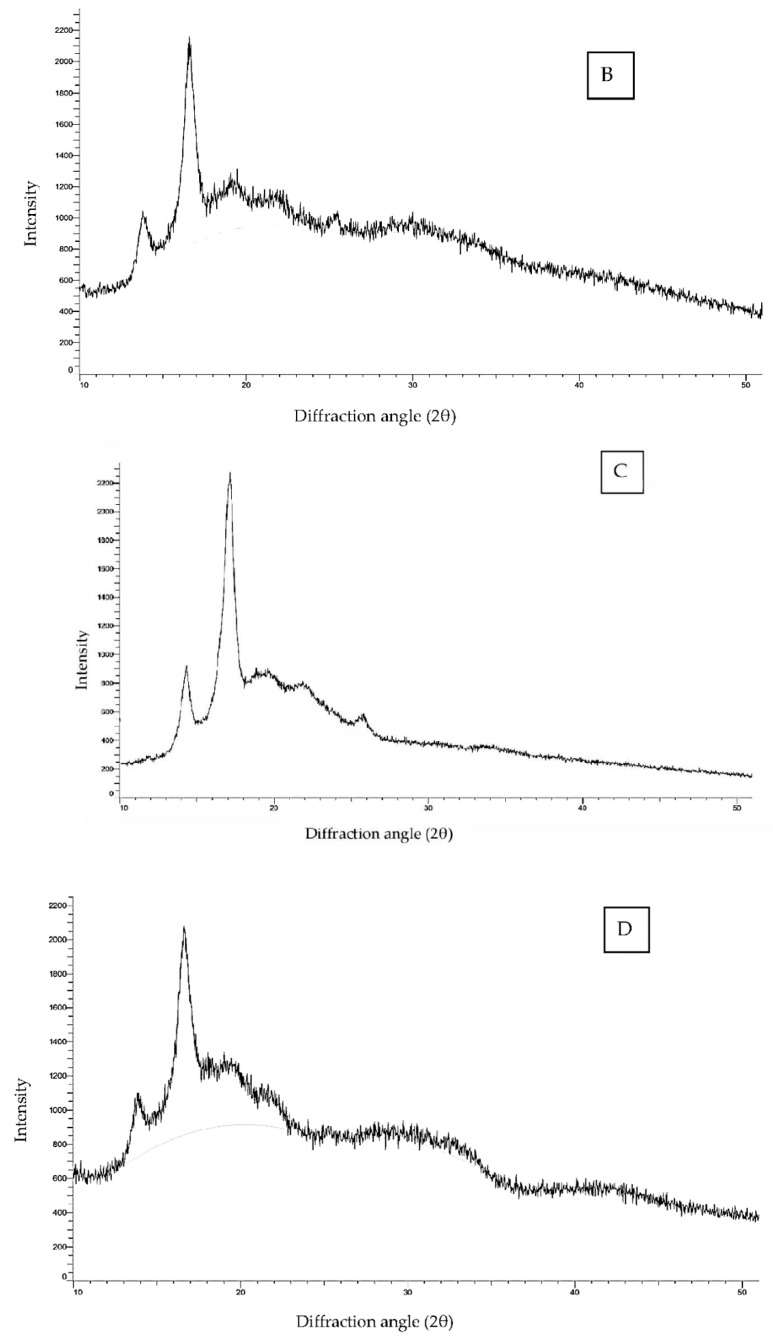
XRD spectra of native and cross-linked lotus seed starch. (**A**) Native LS, (**B**) CL-LS-1, (**C**) CL-LS-3, (**D**) CL-LS-5.

X-ray diffractometry has been used to reveal the presence and characteristics of the crystalline structure of starch granules. Starch has been demonstrated to have four different crystalline patterns using XRD (A, B, C, or V polymorphs). Figure 2A–D show the X-ray diffractograms of native LS and CL-LS at different levels (1, 3, 5%). There were no significant (*p* ≤ 0.05) differences found in the diffraction spectra of native LS and all CL-LS samples. Strong peaks at 14°, 16°, 18°, 21°, and 26° (2θ) showed a CA-type (close to A-type) diffraction pattern. These findings indicated that CL concentrations up to 5% did not significantly change the crystalline spectra of LS. It is suggested that CL typically occurs in amorphous portions of starch granules without affecting the crystalline spectra [40]. The relative crystallinity (RC) of native LS and CL-LS samples are depicted in Table 1. In comparison with native LS, the RC of the CL-LS samples was reduced. CL-LS at 5% showed the lowest value of RC, i.e., 29.01%, and the highest value was observed for native LS, i.e., 30.27%. RC values were slightly reduced as the cross-linking level was increased, perhaps owing to chemical modification, and changes in the crystal structure developed throughout the cross-linking process. These results are in agreement with Chen et al. [41] who observed a reduction in RC values of cross-linked kudzu root starch compared with its native counterpart.

#### 3.1.5. Pasting Parameters

**Figure 3:**
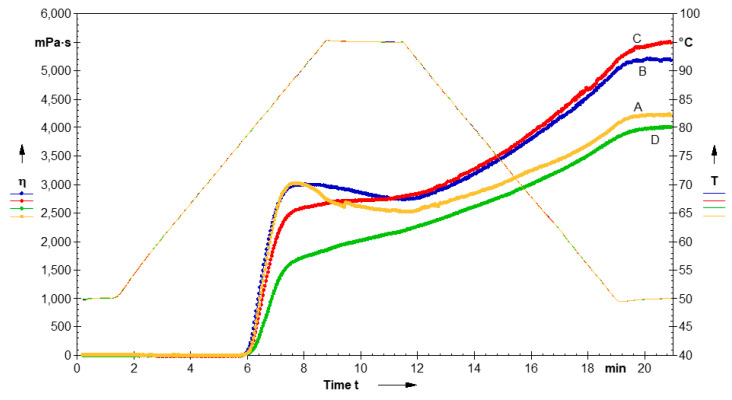
Pasting profile of native and modified cross-linked lotus seed starches. (**A**) Native LS, (**B**) CL-LS-1, (**C**) CL-LS-3, (**D**) CL-LS-5.

The pasting parameters and pasting curves of native LS and CL-LS are shown in Table 2 and Figure 3. During heating and gelatinization, the molecular arrangements of starch undergo a radical alteration, with consequent changes in properties. The pasting temperature (PT) is the minimum temperature required to cook the starch. Peak viscosity (PV) represents the highest viscosity of starches at the equilibrium point between swelling and polymer leaching due to an increase in temperature, while breakdown viscosity (BDV) shows the stability of starch granules against increasing temperatures and shear forces. On cooling, the aggregation of amylose molecules resulting in an increased viscosity is indicated by final viscosity (FV), while setback viscosity (SBV) shows the gelation or retrogradation ability of starch [36]. Native starch showed higher PV (3028.51 MPa·s) in comparison to CL-LS starches (2950.43–1640.22 MPa·s), with CL-LS-5 showing the lowest values. The reduced PV of CL starches may be due to the formation of covalent bonding by cross-linking between amylose and amylopectin, which strengthened the swollen starch granules, thereby lowering the breakage of the starch paste under mechanical and thermal shear [42]. It has been observed that new bond formation in CL starch results in the tightening of starch molecules, thereby preventing their breakage during mechanical and thermal exposure [37]. The BDV of CL-LS at 1% (213.43 MPa·s) was lower in comparison to native LS (362.24 MPa·s), indicating greater thermal stability. The FV of CL-LS was higher at 1 and 3% and subsequently declined at 5% in comparison to native LS. The lower FV of CL-LS-5 may be due to less water absorption leading to the smaller size of the starch granules, consequently exhibiting lower final viscosity development during cooling [42]. CL-LS exhibited a higher PT, varying from 79.52 to 79.7 °C, in comparison to native LS (79.13 °C). The presence of phosphate groups in CL starches may cause an increase in PT by preventing amylose from leaching out of starch molecules, resulting in molecular strengthening and limited swelling during the gelatinization process [43]. According to earlier findings, CL starches can tolerate harsher thermal conditions than native starches during processing such as canning.

#### 3.1.6. Dynamic Rheological Behavior

The rheological behavior of the starch gel plays a vital role in determining the processability, texture, and eating quality of food products prepared using different starches. The experimental data obtained from frequency sweep tests are reported in Table 3. The elastic and viscous nature of the starch gel is indicated by rheological parameters G′ (dynamic storage modulus) and G′′ (loss modulus), respectively. After every deformation cycle, G′ measures the energy recovered while G′′ calculates the energy lost as viscous dissipation of the starch gel [44]. G′ and G′′ values varied between 1784 and 1900 Pa and 136.31 and 144.63 Pa, respectively. The highest and the lowest G′ values were observed for CL 1% and native LS (at 25 °C/6.28 rad/s). The rheological analysis showed G′ *>* G″ for the starch gels of all LS samples (native, modified), which revealed their elastic nature. The magnitude of G′ and G′′ increased with increasing angular frequency, and G′′ showed a more significant increase than G′. After modification, the magnitude of G′ first increased for CL-LS-1 but decreased at a higher concentration of STMP. No cross-over between G′ and G″ was observed, indicating the stability of these starch pastes over the applied frequency range (0.1–100 rad/s). Kim and Yoo [45] have reported that frequency values decrease as the concentration of cross-linking reagents increases.

### 3.2. Properties of Lotus Seed Starch Films

#### 3.2.1. Moisture Content (MC), Thickness, Water Solubility, and Transparency of LS Films

**Figure 4:**
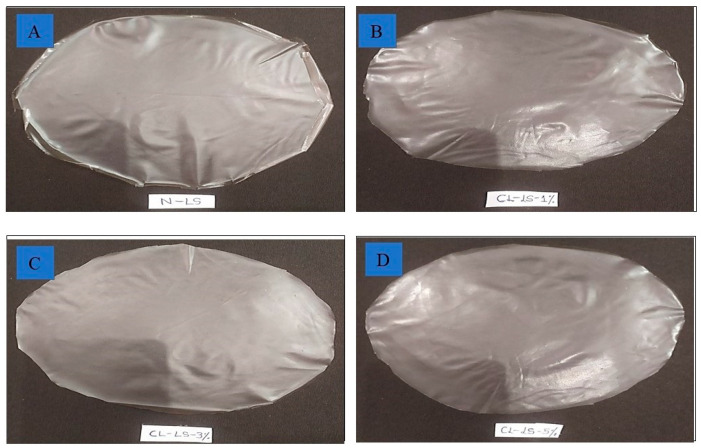
Images of native and modified cross-linked lotus seed starch films. (**A**) Native LSF, (**B**) CL-LSF-1, (**C**) CL-LSF-3, (**D**) CL-LSF-5.

CL significantly (*p* ≤ 0.05) impacted the moisture content (MC) of the CL-LS films (CL-LSFs) tabulated in Table 4 and shown in Figure 4A–D. The MC of the native LS film was 25.12%, followed by CL-LSF with decreased values varying from 20.13 to 23.34% (Table 4). The lowest MC was observed for CL-LSF-5. This may be due to the inclusion of the phosphorus groups which strengthened the hydrogen bridge-type bonds within the starch molecules and reduced the MC of STMP-modified starch films [46].

The thickness of a film is an important characteristic since it is taken into account when calculating the mechanical and barrier properties of a film [47]. Table 4 shows the thickness of the native LSF and CL-LSFs. The thickness varied from 0.10 to 0.15 mm, with the lowest (0.10 mm) observed for the native film, and increased with increasing STMP concentration. Cross-linking strengthens the internal structure of starch molecules, and the incorporation of bulky phosphate groups gives starch molecules a higher molar volume, resulting in an increased CL-LSF thickness [46].

The water solubility (WS) of the film indicates its integrity in an aqueous medium; higher solubility values indicate lower water resistance. The water solubility of the native LSF and CL-LSFs differed significantly (*p* ≤ 0.05). The CL-LSFs showed lower WS (29.23–35.65%) than the native LS film (48.12%). Further, WS decreased with increasing modification levels. A decrease in free hydroxyl with the addition of the CL agents hindered the film matrix affinity for water molecules, resulting in their decreased WS [48]. A similar reduction in WS for CL faba bean starch film was also noticed by Sharma et al. [33].

Transparency is inversely associated with opacity: the lower the opacity value, the higher the transparency of the film [33]. The native LSF and CL-LSFs differed significantly in their opacity values; native LS film exhibited a value of 1.57%, which decreased gradually in the CL-LSFs with increasing modification levels (Table 4). The opacity of starch films depends on the assembling pattern of the starch granules in the films [49] and is influenced by the presence of ordered zones which reduce light absorption and hence increase the transparency of the films. The CL-LSFs exhibited the highest opacity in comparison to the native LS film. The opacity of starch films is associated with the compactness of the matrix [50]. The addition of the phosphate group increased the intermolecular contact between the starch chain and glycerol, resulting in the generation of more compact and opaque CL-LSFs.

#### 3.2.2. Water Vapor Permeability

The water vapor permeability (WVP) of the native LSF and CL-LSFs is shown in Table 4. The highest WVP was observed for the native LS film (1.58 g·m/Pa·s·m^2^). Moreover, the WVP decreased with increasing concentrations of STMP, and the values for the CL-LSFs at the 1, 3, and 5% levels were 1.48, 1.41, and 1.45 g·m/Pa·s·m^2^, respectively. The interaction of starch with the CL agent reduced the availability of hydrophilic groups and limited the starch mobility in amorphous regions, resulting in lower water absorption and improved mechanical properties in the films [5]. Earlier studies also reported improved water barrier properties for CL-starch-based films [5,33,51]. The slight increase in the WVP of CL-LSF-5 could be related to the increased bulkiness and free volume in the film caused by high cross-linking agent concentrations [33].

#### 3.2.3. Mechanical Properties

The maximum stress required during stress–strain experiments, or the force at the sample breakpoint, is referred to as tensile strength (TS). To retain the structural integrity of the food product during storage, shipping, and handling, food packaging must have good mechanical qualities and durability [52]. The effect of CL agents on tensile strength and elongation at break (EB) for LS films is shown in Table 4. The tensile strength of the CL-LSFs was significantly (*p* ≤ 0.05) higher in comparison to the native LS film. The tensile strength of the native LS film was 6.12 MPa, which increased significantly (*p* ≤ 0.05) in the order of CL-LSF-1 (10.45 MPa) < CL-LSF-3 (11.09 MPa) < CL-LSF-5 (12.52 MPa). A rise in CL starch film density due to cross-linkages between hydroxyl groups and STMP might be responsible for this increase in the tensile strength of the CL-LSFs [53]. Moreover, by reacting with the OH- groups of starch, cross-linking agents make starch less hydrophilic, which might account for the increased network resistance of the films. Some earlier studies also confirmed our results of increased tensile strength of CL starch films [33,54].

Elongation at break is the percentage increase in length achieved before the film breaks, which characterizes a film’s capacity to resist changes in shape without breaking, tearing, or cracking. It was observed that the elongation at break of the native LS film was higher than in the CL-LS films. Moreover, raising the concentration level of the CL agents lowered EB from 37.52% to 26.11%. A similar result for the decrease in elongation at break has been observed by Sukhija et al. [55] for lotus rhizome starch composite films at various concentrations of the CL agent. They explained that the incorporation of STMP decreased chain mobility by introducing bulky phosphate groups between starch molecules, which delivered rigidity to the films and hence the elongation at break was decreased.

#### 3.2.4. FTIR Spectral Analysis

**Figure 5:**
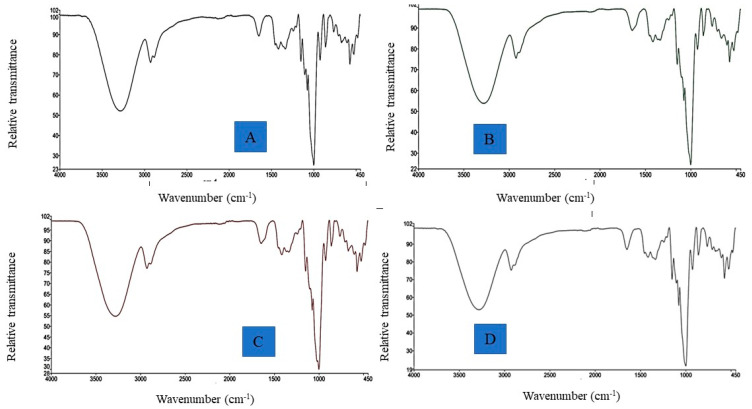
FTIR spectra of native and modified cross-linked lotus seed starch films. (**A**) Native LSF, (**B**) CL-LSF-1, (**C**) CL-LSF-3, (**D**) CL-LSF-5.

A Fourier transform infrared (FTIR) spectroscopy spectrum represents the bands corresponding to stretching, bending, and deformation related to the main functional groups present in the starch granules. The FTIR spectra of the native LSF and CL-LSFs are shown in Figure 5A–D. Four main regions of the FTIR spectra of the LS films contributed to the sequential interpretation and characterization of the key bands, which involved wavenumber ranges of (i) 3000–3600 cm^−1^ (O-H stretch zone), (ii) 2800–3000 cm^−1^ (C-H stretch zone), (iii) 800−1300 cm^−1^ (the fingerprint zone), and (iv) less than 800 cm^−1^. The characteristic bands developed mainly from the vibrational modes of amylopectin and amylose. The Fourier transform infrared spectroscopy spectrograms of all the LS films were typical and identical. For the native LSF and CL-LSFs (1, 3, and 5%), the bands from 3292 cm^−1^ to 3272.19 cm^−1^ were evident for the –OH group stretching vibration. The band from 2930.05 cm^−1^ to 2926.38 cm^−1^ corresponds to the stretching frequency of the C-H group. The presence of absorption bands at 1650.46–1645.18 cm^−1^ ratifies the presence of water. The small peak at 1416.60–1416.25 cm^−1^ might correspond to the -CH_3_ symmetric deformation mode [56]. The peak at 1151.21–1149.59 cm^−1^ corresponds to the polysaccharide (1–4) glycosidic bond stretching vibration [57]. All LS films exhibited absorption bands (at 1000.55–999.02 cm^−1^) that were related to the C-O bond vibrations [37]. The spectral region from 800 to 1150 cm^−1^ might be attributed to the absorption bands of glycerol corresponding to the vibrations of C-C and C-O bonds [58]. Similar spectra of the CL-LS films indicated that CL simply fractured the starch molecules, but the chemical natures remained the same.

#### 3.2.5. Morphological Characteristics

**Figure 6:**
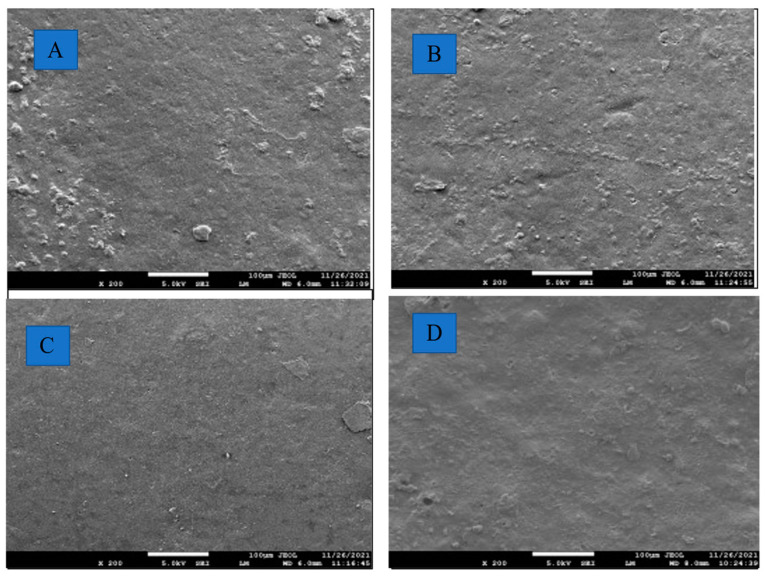
Morphological properties of native and modified cross-linked lotus seed starch films at 200× magnification. (**A**) Native LSF, (**B**) CL-LSF-1, (**C**) CL-LSF-3, (**D**) CL-LSF-5.

A scanning electron microscope (SEM) plays a vital role in examining and comparing the surface characteristics of films. The SEM micrographs of the native LSF and CL-LSFs are presented in Figure 6A–D. The modified LS films showed a more homogeneous and consistent surface in comparison to the native LS film when viewed under a magnification of 200× at 10 kV. It was discovered that the native LS film had the roughest structure, with numerous fractures, holes, and ridges. The CL-LSFs, on the other hand, had better morphological properties, with a smooth surface devoid of fractures, holes, cracks, and ridges. The surface of the film becomes smoother as STMP concentration increases. An earlier study explained that CL agents provide compactness and homogeneity to film structure by drawing the matrix molecules close together, thereby improving the surface smoothness in CL films [59]. CL pearl millet starch films also showed improved surface characteristics with a smooth surface free of cracks, pores, and ridges [5].

#### 3.2.6. Thermal Properties of LS Films (DSC)

The thermal properties of the native LSF and CL-LSFs are shown in Table 5. Starch gelatinization is an irreversible heat-absorbing phase transition in which amylopectin double helices dissociate from a semi-crystalline form to an amorphous structure. It is suggested that starches with low gelatinization temperatures have better cooking characteristics [36]. The gelatinization temperatures (onset T_o_, peak T_p_, conclusion T_c_) of the native LS film were 30.08 °C, 67.30 °C, and 104.82 °C, respectively. The T_o_, T_p_, and T_c,_ of the CL-LSFs varied between 30.19 and 32.47 °C, 74.49 and 83.58 °C, and 122.57 and 127.34 °C, respectively. Compared to the native LSF, the gelatinization endotherms of the CL-LSFs migrated to higher temperatures. The molecular stability of starch granules is known to increase through cross-linking, leading to greater gelatinization temperatures. The CL-LSFs also showed a significant (*p* ≤ 0.05) rise in gelatinization enthalpy (ΔH_gel_) in comparison to their native counterpart. A similar rise in T_o_, T_p_, T_c_, and ΔH_gel_ in CL corn starch films were also reported by Xu et al. [60]. It was concluded that CL results in higher decomposition temperatures due to the formation of a stronger and more compact network of inter- and intramolecular bonds upon the incorporation of CL agents.

## 4. Conclusions

Cross-linking at different concentrations produced significant changes in the thermal, rheological, and physicochemical properties of lotus seed starch. The amylose content, solubility, swelling power, and pasting viscosities declined while the pasting temperature improved after cross-linking of lotus seed starch. The change in different properties gradually increased with increasing STMP levels. Further, the films made from CL-LS were stiffer and more opaque. Cross-linking resulted in higher gelatinization temperatures and enthalpy, which suggests that the starch molecules in cross-linked starch had more compact internal structures. After cross-linking, water solubility and water vapor permeability were both significantly reduced; cross-linking at 5% exhibited the lowest of these values. Moreover, the films produced by 5% cross-linking had greater tensile strength and less elongation at break. Therefore, it can be concluded that LS films’ mechanical and barrier qualities were improved by cross-linking. As a result, these can be utilized in a variety of industrial applications, particularly flexible packaging. Additionally, as lotus seed is an underutilized commodity, it can serve as a suitable replacement for corn starch in starch-based industries.

## Figures and Tables

**Table 1:** Physicochemical properties and relative crystallinity of native and modified starches.

Starch Sample	Degree of Cross-Linking (%)	Amylose Content (%)	Swelling Power (90 °C) (g/g)	Solubility (90 °C) (%)	Relative Crystallinity (%)
Native LS	-	27.98 ± 0.10 ^a^	15.02 ± 0.21 ^a^	16.00 ± 0.13 ^a^	30.27 ± 0.40 ^a^
CL-LS-1	2.58 ± 0.03 ^c^	25.16 ± 0.12 ^b^	14.83 ± 0.19 ^b^	15.44 ± 0.12 ^b^	29.21 ± 0.45 ^b^
CL-LS-3	15.77 ± 0.16 ^b^	19.36 ± 0.13 ^c^	12.44 ± 0.11 ^c^	15.35 ± 0.14 ^bc^	29.15 ± 0.55 ^b^
CL-LS-5	45.84 ± 0.21 ^a^	13.45 ± 0.15 ^d^	9.05 ± 0.23 ^d^	15.13 ± 0.15 ^c^	29.01 ± 0.42 ^b^

CL-LS-1: cross-linking at 1%, CL-LS-3: cross-linking at 3%, CL-LS-5: cross-linking at 5%. ^a–d^: Means ± SD within the column with different lowercase superscripts are significantly different (*p* ≤ 0.05).

**Table 2:** Pasting properties of native and cross-linked lotus seed starch.

Sample	PV (MPa·s)	TV (MPa·s)	BDV (MPa·s)	SBV (MPa·s)	FV (MPa·s)	PT (°C)
Native LS	3028.51 ± 34 ^a^	2666.27 ± 35 ^b^	362.24 ± 17 ^a^	2085.57 ± 31 ^b^	4751.84 ± 40 ^c^	79.13 ± 0.03 ^c^
CL-LS-1	2950.43 ± 33 ^b^	2737 ± 21 ^a^	213.43 ± 12 ^b^	2458 ± 38 ^a^	5195 ± 41 ^b^	79.7 ± 0.07 ^a^
CL-LS-3	2550.80 ± 28 ^c^	-	-	-	5489.24 ± 37 ^a^	79.7 ± 0.06 ^a^
CL-LS-5	1640.22 ± 19 ^d^	-	-	-	4007.86 ± 39 ^d^	79.52 ± 0.06 ^b^

PV: peak viscosity, TV: trough viscosity, BDV: breakdown viscosity, FV: final viscosity, SBV: set back viscosity, PT: pasting temperature. ^a–d^: Means ± SD within the column with different lowercase superscripts are significantly different (*p* ≤ 0.05).

**Table 3:** Dynamic rheological properties of native and modified lotus seed starch.

Sample	Storage Modulus G′ (Pa)	Loss Modulus G″ (Pa)	tanδ (G″/G′)
Native LS	1784 ± 14 ^c^	144.63 ± 3 ^a^	0.08
CL-LS-1	1900 ± 18 ^a^	140.92 ± 2 ^a^	0.07
CL-LS-3	1830 ± 15 ^b^	136.31 ± 3 ^b^	0.07
CL-LS-5	1796 ± 15 ^c^	136.52 ± 2 ^b^	0.07

^a–d^: Means ± SD within the column with different lowercase superscripts are significantly different (*p* ≤ 0.05).

**Table 4:** Moisture content, thickness, water solubility, opacity, water vapor permeability, and mechanical properties of native and cross-linked lotus seed starch films.

Film Sample	Moisture Content (%)	Thickness (mm)	Water Solubility (%)	Opacity (%)	WVP (g·m/Pa·s·m^2^)	Tensile Strength (MPa)	Elongation at Break (%)
Native LSF	25.12 ± 0.16 ^a^	0.10 ± 0.00 ^c^	48.12 ± 0.21 ^a^	1.57 ± 0.01 ^d^	1.58 ± 0.01 ^a^	6.12 ± 0.12 ^d^	37.52 ± 1.34 ^a^
CL-LSF-1	23.34 ± 0.20 ^b^	0.13 ± 0.01 ^b^	35.65 ± 0.19 ^b^	2.59 ± 0.02 ^a^	1.48 ± 0.03 ^b^	10.45 ± 0.15 ^c^	35.54 ± 1.52 ^a^
CL-LSF-3	22.22 ± 0.14 ^c^	0.14 ± 0.02 ^ab^	33.45 ± 0.16 ^c^	2.27 ± 0.01 ^b^	1.41 ± 0.05 ^c^	11.09 ± 0.13 ^b^	30.91 ± 0.88 ^b^
CL-LSF-5	20.13 ± 0.22 ^d^	0.15 ± 0.01 ^a^	29.23 ± 0.11 ^d^	2.07 ± 0.02 ^c^	1.45 ± 0.02 ^bc^	12.52 ± 0.14 ^a^	26.11 ± 0.52 ^c^

WVP: water vapor permeability, CL-LSF-1: cross-linked lotus seed starch film at 1% STMP, CL-LSF-3: cross-linked lotus seed starch film at 3% STMP, CL-LSF-5: cross-linked lotus seed starch film at 5% STMP. ^a–d^: Means ± SD within the column with different lowercase superscripts are significantly different (*p* ≤ 0.05).

**Table 5:** Thermal properties of native and cross-linked lotus seed starch films.

Sample	T_o_ (°C)	T_p_ (°C)	T_c_ (°C)	ΔH_gel_ (J/g)
Native LSF	30.08 ± 0.9 ^b^	67.30 ± 0.11 ^d^	104.82 ± 0.17 ^d^	152.70 ± 0.18 ^d^
CL-LSF-1	32.04 ± 0.3 ^a^	74.49 ± 0.21 ^c^	122.57 ± 0.14 ^c^	177.91 ± 0.22 ^c^
CL-LSF-3	30.19 ± 0.1 ^b^	76.78 ± 0.19 ^b^	123.06 ± 0.16 ^b^	187.47 ± 0.13 ^b^
CL-LSF-5	32.47 ± 0.5 ^a^	83.58 ± 0.18 ^a^	127.34 ± 0.15 ^a^	214.16 ± 0.27 ^a^

T_o_: onset temperature, T_p_: peak temperature, T_c_: conclusion temperature, ΔH_gel_: enthalpy of gelatinization (dwb, based on starch weight). ^a–d^: Means ± SD within the column with different lowercase superscripts are significantly different (*p* ≤ 0.05).

## Data Availability

The data presented in this study is available in this article.
